# The Pharmacological Chaperone AT2220 Increases the Specific Activity and Lysosomal Delivery of Mutant Acid Alpha-Glucosidase, and Promotes Glycogen Reduction in a Transgenic Mouse Model of Pompe Disease

**DOI:** 10.1371/journal.pone.0102092

**Published:** 2014-07-18

**Authors:** Richie Khanna, Allan C. Powe, Yi Lun, Rebecca Soska, Jessie Feng, Rohini Dhulipala, Michelle Frascella, Anadina Garcia, Lee J. Pellegrino, Su Xu, Nastry Brignol, Matthew J. Toth, Hung V. Do, David J. Lockhart, Brandon A. Wustman, Kenneth J. Valenzano

**Affiliations:** Amicus Therapeutics Inc., Cranbury, New Jersey, United States of America; Hertie Institute for Clinical Brain Research and German Center for Neurodegenerative Diseases, Germany

## Abstract

Pompe disease is an inherited lysosomal storage disorder that results from a deficiency in acid α-glucosidase (GAA) activity due to mutations in the *GAA* gene. Pompe disease is characterized by accumulation of lysosomal glycogen primarily in heart and skeletal muscles, which leads to progressive muscle weakness. We have shown previously that the small molecule pharmacological chaperone AT2220 (1-deoxynojirimycin hydrochloride, duvoglustat hydrochloride) binds and stabilizes wild-type as well as multiple mutant forms of GAA, and can lead to higher cellular levels of GAA. In this study, we examined the effect of AT2220 on mutant GAA, *in vitro* and *in vivo*, with a primary focus on the endoplasmic reticulum (ER)-retained P545L mutant form of human GAA (P545L GAA). AT2220 increased the specific activity of P545L GAA toward both natural (glycogen) and artificial substrates *in vitro*. Incubation with AT2220 also increased the ER export, lysosomal delivery, proteolytic processing, and stability of P545L GAA. In a new transgenic mouse model of Pompe disease that expresses human P545L on a *Gaa* knockout background (Tg/KO) and is characterized by reduced GAA activity and elevated glycogen levels in disease-relevant tissues, daily oral administration of AT2220 for 4 weeks resulted in significant and dose-dependent increases in mature lysosomal GAA isoforms and GAA activity in heart and skeletal muscles. Importantly, oral administration of AT2220 also resulted in significant glycogen reduction in disease-relevant tissues. Compared to daily administration, less-frequent AT2220 administration, including repeated cycles of 4 or 5 days with AT2220 followed by 3 or 2 days without drug, respectively, resulted in even greater glycogen reductions. Collectively, these data indicate that AT2220 increases the specific activity, trafficking, and lysosomal stability of P545L GAA, leads to increased levels of mature GAA in lysosomes, and promotes glycogen reduction *in situ*. As such, AT2220 may warrant further evaluation as a treatment for Pompe disease.

## Introduction

Pompe disease (acid maltase deficiency, glycogen storage disease type II; OMIM 232300) is a lysosomal storage disorder caused by mutations in the gene (*GAA*) that encodes the lysosomal hydrolase acid α-glucosidase (GAA) [1]–[2]. Deficiency in GAA activity results in glycogen accumulation and deposition in the lysosomes of heart, skeletal muscle, and other tissues [3]. Pompe disease shows a broad phenotypic spectrum that ranges from the severe infantile-onset form to more slowly progressing, later-onset forms. Infantile-onset Pompe disease patients have little or no GAA activity, present with hypotonia, cardiomegaly, and cardiorespiratory distress, and typically die by age 2 if untreated [4]. Late-onset forms of Pompe disease typically show some detectable GAA activity, present in childhood or adulthood, and progress more slowly, with musculoskeletal and pulmonary involvement leading to progressive weakness and respiratory insufficiency [5].

Enzyme replacement therapy (ERT) currently is the only approved treatment for Pompe disease, administered as a bi-weekly intravenous infusion of recombinant human GAA (rhGAA; alglucosidase alfa, marketed as Myozyme and Lumizyme, Genzyme Corp., a Sanofi company, Cambridge, MA). Treatment with rhGAA improves cardiac function, motor skills, and life span in infantile-onset patients [6]–[11], and leads to mild improvements in motor and respiratory function in late-onset patients [12]–[13]. Despite the clinical benefits of ERT, the efficacy of rhGAA may be limited by insufficient targeting and uptake into key disease-relevant tissues, as well as poor tolerability due to immunogenic and anaphylactic reactions to the exogenous enzyme [9], [13]–[18]. In addition, ERT does not address the neurological manifestations associated with Pompe disease since rhGAA does not cross the blood-brain barrier. Hence, a clear unmet medical need still exists for many Pompe patients.

GAA is synthesized as a 110 kDa immature glycoprotein precursor in the endoplasmic reticulum (ER), and undergoes a series of proteolytic and *N*-glycan processing events to yield the 95 kDa intermediate and the 76 and 70 kDa mature isoforms [19]–[21]. Processing into the intermediate and mature species occurs in late endosomes and lysosomes, respectively [19], with the final mature isoforms showing significantly increased affinity and activity towards glycogen compared to the precursor forms [20], [22]. Hence, increasing the amount of mature, active GAA in lysosomes is an important step toward the goal of increasing glycogen hydrolysis and reducing substrate accumulation in this disease.

Small molecule pharmacological chaperones (PCs) have been proposed as a potential alternative to ERT for the treatment of Pompe disease [23]–[26]. PCs are thought to selectively bind and stabilize enzymes during synthesis in the ER, facilitating proper protein folding and trafficking, and increasing lysosomal levels and activity [25], [27]–[29]. We and others have shown that the iminosugar 1-deoxynojirimycin (DNJ) hydrochloride (AT2220, duvoglustat hydrochloride) can selectively bind and stabilize multiple mutant forms of GAA, thereby facilitating proper protein folding and increasing the cellular levels of mature GAA, indicative of improved trafficking to lysosomes [25]–[26]. Similarly, *N*-butyl-DNJ was also shown to increase the cellular levels of mature mutant GAA [24]–[25]. However, these earlier studies did not delineate the points during GAA synthesis and maturation at which these PCs act, nor did they demonstrate increases in lysosomal GAA activity as measured by reduced glycogen levels *in situ*.

Here we demonstrate that AT2220 has multiple modes of action during the synthesis and maturation of mutant GAA, including increased catalytic activity prior to proteolytic processing in lysosomes, facilitated export from the ER with subsequent trafficking and processing through the secretory pathway to lysosomes, and stabilization of the mature isoforms in lysosomes. Furthermore, to study the *in vivo* effects of AT2220, a new mouse model of Pompe disease was created. These mice express a human transgene of mutant P545L GAA on a *Gaa* knockout background (hP545L GAA Tg/KO), and show low GAA activity and elevated glycogen levels in disease-relevant tissues including heart, diaphragm, multiple skeletal muscles, and brain. Daily oral administration of AT2220 to hP545L GAA Tg/KO mice resulted in significant and dose-dependent increases in GAA activity with concomitant reduction in tissue glycogen levels; less-frequent AT2220 administration regimens resulted in even greater glycogen reduction. Collectively, these results provide support for the continued evaluation of AT2220 as a potential treatment for Pompe disease. Furthermore, unlike pure *Gaa* knockout mice, the P545L GAA Tg/KO mice were tolerant to repeat administrations of rhGAA, potentially providing a useful model for long-term preclinical ERT studies.

## Materials and Methods

### Materials

AT2220 (1-deoxynojirimycin hydrochloride, duvoglustat hydrochloride) was synthesized by WuXi PharmaTech (Shanghai, China). All cell culture reagents were purchased from Invitrogen (Carlsbad, CA), except for characterized fetal bovine serum (FBS), which was purchased from HyClone (Waltham, MA). Wild-type (CRL-2076) fibroblasts were purchased from ATCC (Manassas, VA); Pompe P545L fibroblasts were a kind gift of Dr. W. J. Kleijer (Department of Cell Biology and Genetics, Erasmus University, Rotterdam, Netherlands); Pompe patient R854X fibroblasts were purchased from Coriell Institute (Camden, NJ). The rabbit anti-human GAA polyclonal antibody, FL059, was a kind gift of Dr. Barry Byrne (University of Florida, Gainesville). Horseradish peroxidase-conjugated (HRP) goat anti-rabbit IgG secondary antibody was purchased from Thermo Fisher Scientific (Rockford, IL). The rat anti-mouse LAMP1 antibody was purchased from Abcam (Cambridge, MA). *Gaa* knockout (KO) mice on a pure 129SVE background were kindly provided by Dr. Barry Byrne. Homozygous transgenic (Tg) mice that express a mutant form of human GAA (hP545L) on a *Gaa* KO background (hP545L GAA Tg/KO) and wild-type littermates were generated as described below. Animal husbandry and experiments were conducted under Rutgers University Institutional Animal Care and Use Committee approved protocols. The Rutgers animal facility has maintained accreditation with the Association for the Assessment and Accreditation of Laboratory Animal Care-International since July 8, 1994. Rutgers University maintains an Assurance with the Office of Laboratory Animal Welfare (#A3262-01). All other reagents were purchased from Sigma Aldrich (St. Louis, MO) unless noted otherwise.

### Cell culture

Wild-type fibroblasts, Pompe patient-derived fibroblasts, and transfected COS-7 cells were cultured and treated as described in Flanagan, *et al*. (2009) [26].

### Measurement of GAA activity in lysed cells

GAA activity was measured using the fluorgenic substrate 4-methylumbelliferyl-α-D-glucopyranoside (4-MUG; 3 mM) as described previously [26]. To measure GAA enzyme activity using glycogen as a substrate, lysates from transfected COS-7 cells were prepared as described previously [26]. GAA-mediated hydrolysis of glycogen was measured in a two-step process. First, approximately 5 mg total cellular protein was incubated with 100 mg rabbit liver glycogen in 50 mM potassium acetate, pH 4.0 (final volume 10 mL), for 3 hours. First-stage reactions were stopped with 1 mL 0.5 M NaOH. Second, liberated glucose was measured using a glucose oxidase/peroxidase-coupled assay method according to the manufacturer's instructions (Amplex Red Glucose Detection, Invitrogen). Enzyme activity is expressed as nanomoles of glucose released per milligram cellular protein per hour (nmol glucose/mg protein/hr) as determined using a glucose standard curve.

### Western blot analysis

GAA protein levels in lysates from fibroblasts and transfected COS-7 cells were analyzed by SDS-PAGE/immunoblotting after adding equivalent amounts of total protein per lane and used β-actin as a loading control as described previously [26].

### Concanavalin A pulldowns of GAA from conditioned media

Upon collection, conditioned media (15 mL) from transfected COS-7 cells were pH adjusted with 100 mM Bis-Tris, pH 6.5, and concentrated 15-fold using 30,000 MW cutoff filters (Centricon; Millipore, Billerica, MA) to a final volume of 1 mL. Approximately 1 mL conditioned medium was incubated with 50 µL Concanavalin A-Sepharose (50% slurry; GE Healthcare, Piscataway, NJ) pre-equilibrated with Lysis Buffer (150 mM NaCl, 0.1% Triton X-100, 25 mM Bis-Tris, pH 6.5). The mixture was incubated with rocking for at least 1 hour at 4°C. Beads were collected by centrifugation and washed thrice with Lysis Buffer. Half the beads were used for enzyme activity assays and the other half for SDS-PAGE/immunoblotting as described previously [26].

### Immunofluorescence of GAA in transfected COS-7 cells

COS-7 cells were plated at 70,000 cells per well in 12-well coverslip-bottomed plates (MatTek Corporation, Ashland, MA) and were cultured, transfected, and incubated with AT2220 as described above. Two to three days post-transfection, cells were fixed with 3.7% paraformaldehyde/0.63× Dulbecco's PBS (D-PBS), permeabilized with 0.5% saponin in 1× D-PBS, and blocked with 0.1% bovine serum albumin (BSA), 0.1% saponin, 1× D-PBS. Fixed, permeabilized cells were incubated with a mouse anti-human GAA monoclonal antibody (1∶200; kind gift of Dr. J. LeBowitz, Biomarin, Novato, CA) and a rabbit anti-human calnexin polyclonal antibody (1∶200; Abcam) in 0.1% BSA, 0.1% saponin, 1× D-PBS for 1 hour at room temperature, washed with 0.1% BSA, 0.1% saponin, 1× D-PBS, then incubated with secondary antibodies (donkey anti-mouse Alexa 594 and donkey anti-rabbit Alexa 488, respectively, both 1∶500; Invitrogen) for 1 hour at room temperature. Images of stained cells were acquired using a 40× (1.3 NA) oil immersion objective and a 12-bit CCD camera (Photometrics CoolSnap ES2, Tuscon, AZ) coupled to a Nikon TE2000 microscope controlled with NIS Elements AR software (Melville, NY).

### Metabolic labeling and immunoprecipitation of GAA

Fibroblasts and transfected COS-7 cells in 6-well plates were labeled for the indicated periods of time with cysteine, methionine-free medium (Delbecco's Modified Eagle Medium (DMEM), 10% FBS, 25 mM HEPES, pH 7.4) supplemented with ^35^S-cysteine and methionine (100 µCi EXPRE^35^S^35^S per well; Perkin-Elmer, Waltham, MA). At the end of the pulse period, the labeling medium was replaced with complete DMEM for the indicated chase period. Labeled fibroblasts and transfected COS-7 cells were trypsinized, collected by centrifugation, lysed in 1 mL 150 mM NaCl, 0.1% Triton X-100, 0.2% SDS, 25 mM Bis-Tris, pH 6.5 (IP Buffer), and clarified by centrifugation. Clarified lysates were precleared with 50 µL Ultra-Link Protein G beads (50% slurry; Pierce, Rockford, IL) pre-equilibrated with IP Buffer. Pre-cleared lysates were then incubated with 2 µL rabbit anti-human GAA primary antibody FL059 (1∶1000 dilution) and 50 µL Ultra-Link Protein G beads in IP Buffer plus 0.2% BSA for 1 hour at room temperature. Beads were collected by centrifugation and washed thrice with IP buffer. Immunoprecipitated GAA was eluted by addition of 4× LDS (Invitrogen) and denatured at 70°C for 10 minutes. Eluates were electrophoresed using SDS-PAGE (4–12% Bis-Tris NuPAGE, 1× MOPS buffer, 180 V for 1 hour; Invitrogen). Gels were fixed, dried, imaged using a Storm PhosphorImager (GE Healthcare, Piscataway, NJ), and analyzed with the manufacturer's densitometry software. Densitometry data were fitted to a one-phase exponential decay function using Prism (GraphPad, La Jolla, CA) to estimate the half-lives of GAA isoforms.

### Generation of hP545L GAA Tg/KO mice

The hP545L GAA Tg/KO mice were generated as described previously for Fabry Tg/KO mice [30]–[32]. Briefly, the P545L mutation was introduced into the human *GAA* gene using a PCR mutagenesis kit (Clontech Laboratories Inc., Mountain View, CA), and cloned into a pCI mammalian expression vector (Promega Corporation, Madison, WI) containing a CMV promoter. The construct was used to generate founder mice via routine methodologies described previously [33]. The transgenic founder mice were bred first with wild-type C57BL/6 mice to obtain the transgenic F1 generation on a wild-type *Gaa* background, and finally with *Gaa* KO mice to obtain the hP545L GAA Tg/KO mice. For confirmation of the transgenic allele, a 500 base pair fragment was amplified using 5′- TACGTATTAGTCATCGCTAT -3′ forward and 5′- ATTAAGTACTCTAGCCTTAA -3′ reverse primers. The PCR amplification consisted of 30 cycles of denaturation at 94°C for 30 seconds, annealing at 55°C for 1 minute, and elongation at 72°C for 2 minutes. The KO allele was confirmed using methodology as described previously [34].

### Administration of AT2220

AT2220 was orally administered to mice *ad libitum*, with the appropriate concentrations of AT2220 in drinking water determined based on the average daily consumption of hP545L GAA Tg/KO mice (approximately 5 mL water/day per mouse). Dosing solutions were made fresh weekly. All doses represent the free base equivalent of the hydrochloride salt form. At study completion, mice were euthanized with CO_2_ and body weights were recorded. Whole blood was drawn into lithium heparin tubes from the inferior vena cava, and plasma was collected by centrifuging blood at 2700 g for 10 minutes at 4°C. Heart, diaphragm, tongue, skin (shaved and removed from the lower ventral side of the neck), hindlimbs (for isolation of quadriceps and gastrocnemius), and forelimbs (for isolation of triceps and biceps) were quickly removed, rinsed in cold phosphate-buffered saline, blotted dry, and stored on dry ice. Tissue samples were also stored in fixatives as described below for immunohistochemical analyses.

### Measurement of tissue GAA levels

Homogenates were prepared by pulsing approximately 50 mg tissue for 3 to 5 seconds on ice with a micro-homogenizer (Pro Scientific, Thorofare, NJ) in 200 µL Lysis Buffer. Homogenates (20 µL) were added to 50 µL Assay Buffer (3 mM 4-MUG in 50 mM potassium acetate, pH 4.0) as described previously [35]. A Micro BCA Protein Assay (Pierce) was used to determine total protein concentration in homogenates according to the manufacturer's instructions. A 4-methylumbelliferone (4-MU) standard curve ranging from 1.3 nM to 30 µM was run in parallel each day for conversion of fluorescence data to absolute GAA activity expressed as nmol 4-MU released per mg total protein per hour (nmol/mg protein/hr). For Western blotting, lysates (approximately 50 µg total protein) were subjected to SDS-PAGE on 12% polyacrylamide gels (Bio-Rad, Hercules, CA), transferred to PVDF membranes (Bio-Rad), and immunoblotted with the rabbit anti-human GAA primary antibody FL059 (1∶1000 dilution) using methods described previously [35].

### Measurement of tissue glycogen levels

Glycogen levels were measured as described previously following homogenization of approximately 50 mg tissue for 3 to 5 seconds on ice with a micro-homogenizer in 200 µL Lysis Buffer [35].

### Histochemical detection of glycogen

Various fixation and processing/embedding procedures were employed prior to tissue glycogen staining. Heart and gastrocnemius were fixed in a mixture of 4% formaldehyde/90% ethanol and embedded in paraffin; skeletal muscles were fixed in 3% glutaraldehyde and embedded in Epon; brain and spinal cord were fixed in neutral-buffered formalin (NBF), post-fixed in 1% periodic acid/NBF, and embedded in paraffin as described previously [36]. Glycogen staining was carried out on paraffin sections (5 µm) and Epon sections (2 µm) using a Periodic Acid-Schiff (PAS) Kit (Sigma) according to the manufacturer's instructions as described previously [35]–[36]. Sections were counterstained in hematoxylin, Richardson's stain, or Luxol Fast Blue, and mounted in Acrytol (Surgipath Medical Industries, Richmond, IL).

### Immunohistochemical (IHC) staining of LAMP1

Tissues were fixed in Z-Fix (Anatech LTD, Battle Creek, MI) and embedded in paraffin. Sections (5 µm) were dewaxed and rehydrated in deionized water. The endogenous peroxidase activity and non-specific background were blocked with 3% H_2_O_2_ (Fisher, Pittsburgh, PA) and Rodent Block M (Biocare Medical, Concord, CA), respectively. After an overnight incubation with a rat anti-mouse LAMP1 antibody (1∶500 dilution) at 4°C, sections were incubated with components from the Promark rat-on-mouse HRP-polymer kit (Biocare Medical); stain was developed with a Betazoid DAB kit (Biocare Medical).

### Tissue and plasma AT2220 quantitation

Mouse tissues were homogenized and extracted (300 µL water per 50 mg tissue) using a Fast Prep homogenizer (MP Biomedical, Irvine, CA) followed by solid phase extraction (SPE). Briefly, 100 µL tissue homogenate or plasma was spiked with 50 ng/mL ^13^C_6_-AT2220 internal standard (manufactured by MDS Pharma Services, Lincoln, NE). Each sample was vortexed and centrifuged at 10,000 g for 5 minutes at room temperature. Supernatants were transferred to a 96-well SPE plate conditioned with methanol, and washed with 0.3% ammonium hydroxide in methanol/water (90∶10), and eluted with 250 µL 1% ammonium hydroxide in methanol/water (90∶10). Samples were dried and finally reconstituted with 100 µL 95∶5∶0.05 acetonitrile (ACN):water:formic acid and 30 µL was used to determine the AT2220 levels by liquid chromatography-tandem mass spectrometry (LC: Shimadzu, Columbia, MD; MS/MS: Sciex API 4000 MS/MS, Applied Biosystems, Foster City, CA). The LC was conducted using an ACN:water:formate binary mobile phase system (mobile phase A: 5 mM ammonium formate, 0.05% formic acid in 95∶5 ACN:water; mobile phase B: 5 mM ammonium formate, 0.05% formic acid in 55∶45 ACN:water) with a flow rate of 0.6 mL/minute on an amide-80 column (50×2 mm, 5 µm) (Tosoh Bioscience). The MS/MS analysis was carried out under atmospheric pressure chemical ionization positive ion mode. The following transitions were monitored: mass/charge (m/z) 164.01→m/z 79.90 for AT2220 and m/z 170.09→m/z 134.0 for the internal standard. An 11-point calibration curve and quality control samples were prepared in the same manner as the samples. The ratio of the area under the curve for AT2220 to that of the internal standard was determined, and final concentrations of AT2220 in each sample were calculated using the linear least squares fit equation applied to the calibration curve. To derive approximate molar concentrations, one gram of tissue was estimated as one mL of volume.

### Data analysis

Determinations of statistical significance were conducted using Microsoft Excel 2003 (Redmond, WA) or GraphPad Prism version 5 (San Diego, CA) as defined in the figure and table legends. Linear trends for dose dependence were calculated using a one-way ANOVA in GraphPad Prism.

## Results

### AT2220 increases the specific activity and lysosomal trafficking of mutant GAA

**Figure 1:**
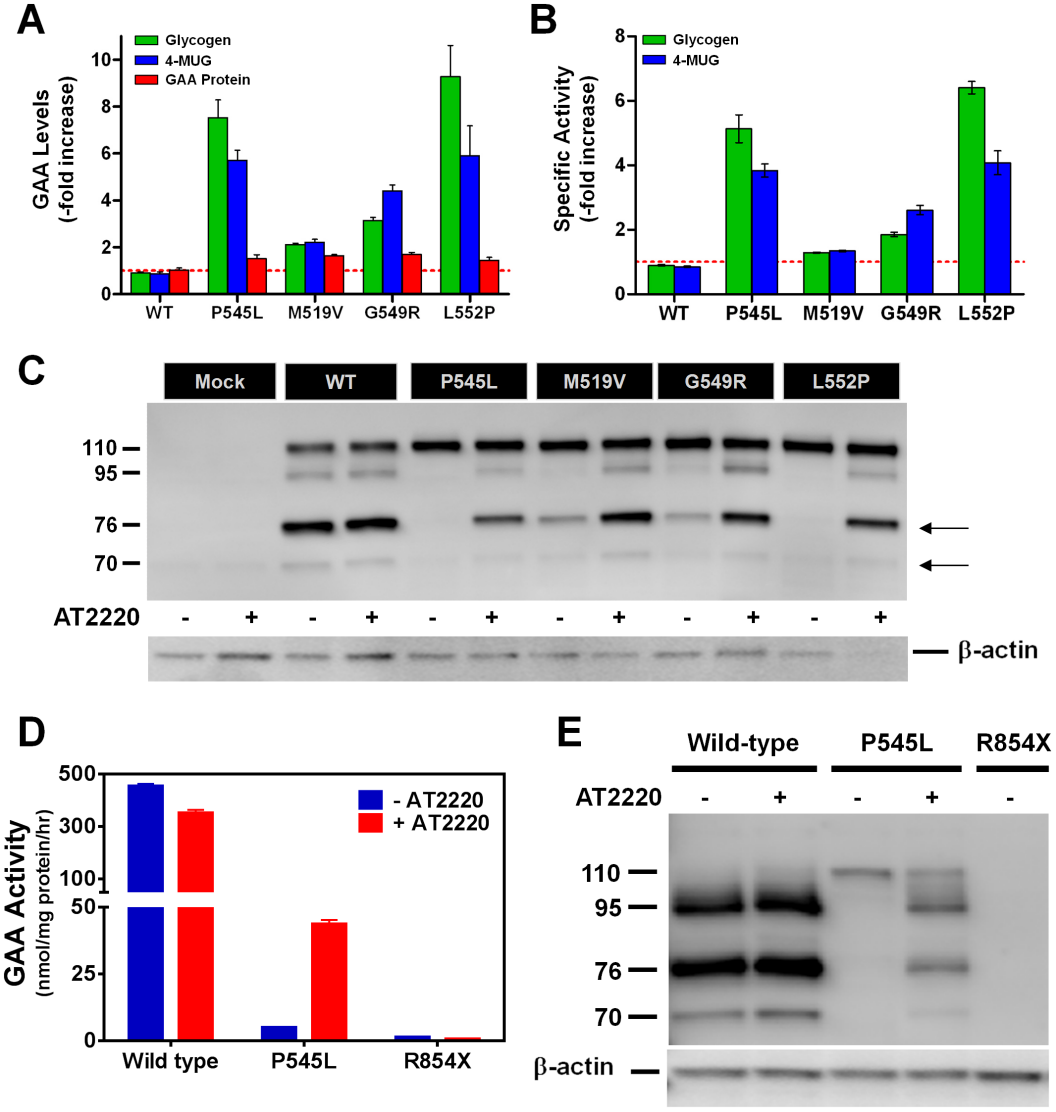
AT2220 increases the specific activity, processing, and lysosomal delivery of multiple mutant forms of GAA. (**A**) AT2220-mediated increases in GAA activity and protein levels in lysates from COS-7 cells transiently transfected with different mutant forms of GAA. Transfected cells were incubated with 100 µM AT2220 for 3 days, harvested, and lysed for activity and protein measurements. GAA activity was measured using the natural substrate glycogen (green) or the artificial substrate 4-methylumbelliferyl-α-glucopyranoside (4-MUG; blue). GAA (all isoforms) were detected through immunoblotting using the rabbit polyclonal antisera FL059 (see also, **Fig. 1C**). GAA protein levels were then quantified by densitometry using known amounts of rhGAA as calibration standards. The red dotted line indicates baseline (*i.e*., no change). (**B**) AT2220-mediated increases in the relative specific activities of multiple mutant forms of GAA calculated as the ratio of GAA enzyme activity against glycogen or 4-MUG to the GAA protein levels in transfected COS-7 cells. Enzyme activity and protein levels were measured as described in **Fig. 1A**. The red dotted line indicates no change from baseline. (**C**) AT2220-dependent increases in the processing and maturation of different mutant forms of GAA. Transfected cells were incubated with or without 100 µM AT2220, harvested, lysed, and immunoblotted as described in **Fig. 1A**. Dashes on the left of the Western blot represent the molecular weights (kDa) of the different GAA isoforms; arrows on the right indicate the presence of the mature, lysosomal 76 and 70 kDa forms of GAA. (**D** and **E**) Human skin fibroblasts from normal and Pompe subjects were incubated for 5 days with or without 100 µM AT2220. Cells were then harvested, lysed, and assayed for GAA activity using 4-MUG (**D**), and for GAA protein levels by immunoblotting (**E**). Dashes on the left of the Western blot represent the molecular weights (kDa) of the different GAA isoforms. WT, wild-type.

Previous studies indicated that the iminosugar 1-deoxynojirimycin (AT2220; duvoglustat hydrochloride) acts as a pharmacological chaperone for various mutant forms of acid α-glucosidase (GAA) [26]. To determine how AT2220 increases cellular GAA activity, COS-7 cells were transfected with various human GAA mutants (P545L, M519V, G549R, and L552P) followed by 3-day incubation with 100 µM AT2220. Cells were then harvested, lysed, and assessed for GAA protein levels by Western blot, and enzymatic activity using either glycogen or 4-MUG as the substrate. As described previously [26], cellular levels of GAA activity against the 4-MUG substrate increased several-fold for the mutant forms of GAA that were tested (**Fig. 1A**). Cellular levels of GAA activity toward the natural substrate glycogen also increased several-fold (**Fig. 1A**). In contrast, cellular GAA protein levels were increased less than 2-fold (**Fig. 1A**). The ratio of cellular GAA activity levels to cellular GAA protein levels gives a measure of the specific activity of the GAA mutants. Incubation with AT2220 increased the specific activity of P545L and L552P GAA 4- to 6-fold against both substrates; the increase for G549R was approximately 2-fold; M519V was unchanged (**Fig. 1B**). These results suggest that AT2220 increases cellular activity, in part by improving the specific activity of some mutant forms of GAA against both natural and artificial substrates.

Previous studies also indicated that AT2220 promotes the processing of GAA mutants from precursor (110 kDa) to the mature lysosomal (70 and 76 kDa) forms [25]–[26]. To determine if AT2220-mediated increases in GAA processing were responsible for the improvements in enzymatic activity, the isoform distribution of GAA mutants synthesized in the absence and presence of AT2220 was examined by Western blotting (**Fig. 1C**). In the absence of AT2220, cells transfected with P545L and L552P showed only the precursor form; incubation with AT2220 resulted in the appearance of the intermediate (95 kDa) and the mature lysosomal forms of the protein (**Fig. 1C**). For the M519V and G549R mutants, incubation with AT2220 increased the levels of mature GAA isoforms already present in untreated cells. Taken together with the activity data, these results suggest that the AT2220-mediated increases in enzymatic activity correlate with increased levels of the mature, lysosomal isoforms.

To verify the results obtained using heterologous expression of GAA, skin fibroblasts derived from normal and Pompe subjects homozygous for either P545L or R854× GAA were incubated for 5 days with 100 µM AT2220. In P545L fibroblasts, AT2220 incubation resulted in a 6-fold increase in cellular GAA activity as measured using 4-MUG, and a 2-fold increase in GAA protein levels as measured by Western blot, leading to a 3-fold increase in specific activity (**Fig. 1D and 1E**). AT2220 also increased the hydrolytic activity towards glycogen in P545L fibroblast lysates (data not shown). Furthermore, AT2220 incubation resulted in the appearance of the mature lysosomal GAA isoforms in P545L fibroblasts (**Fig. 1E**). These results corroborate results seen in the COS-7 heterologous expression experiments with P545L GAA. No GAA activity or protein was seen in R854× fibroblasts in the absence or presence of AT2220.

### AT2220 increases the specific activity of mutant GAA independent of processing

**Figure 2:**
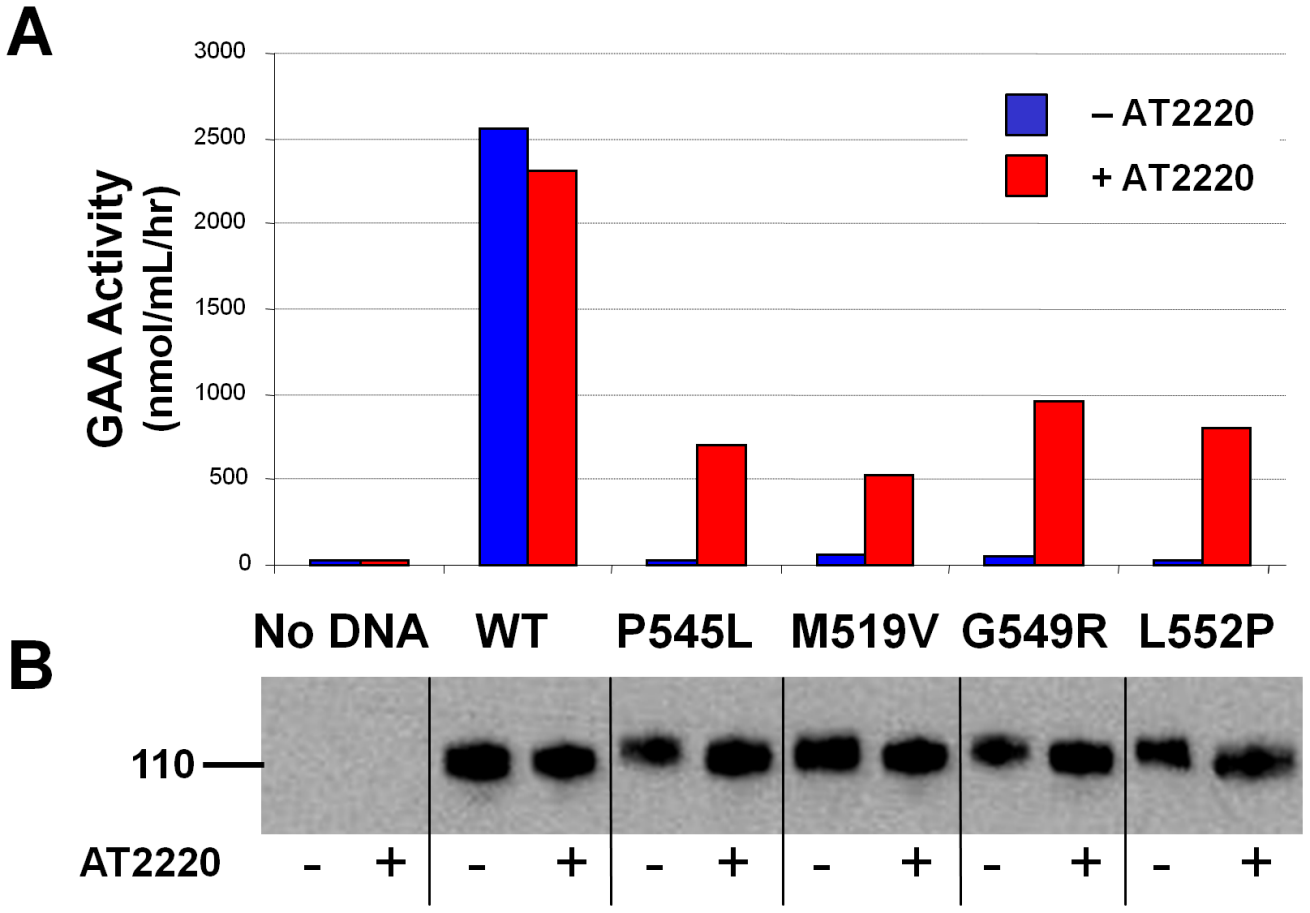
AT2220 increases the specific activity of mutant GAA precursors. (**A**) Enzymatic activity of mutant GAA secreted from COS-7 cells transiently expressing GAA mutants and incubated with or without 100 µM AT2220. GAA in conditioned media was collected by incubation with Concanavalin A-Sepharose (Con-A) beads followed by centrifugation for GAA enrichment and removal of AT2220. Enzymatic activity was measured on half of the beads using the artificial substrate 4-MUG. (**B**) Immunoblotting of conditioned media from COS-7 cells transfected with different GAA mutants. GAA precursor protein (the 110 kDa species) in the remaining half of the Con-A pulldowns was denatured with sample buffer, separated by SDS-PAGE, immunoblotted, and quantified as described in **Fig. 1**. WT, wild-type.

Previous studies indicated that processing of GAA from precursor to its mature lysosomal forms was responsible for increased catalytic activity toward the natural substrate glycogen, but not the artificial substrate 4-MUG [20]. To determine if the AT2220-mediated increases in enzymatic activity of the GAA mutants were dependent on proteolytic processing, we examined the activity of overexpressed wild-type and mutant GAA *precursors* that were secreted from transfected COS-7 cells [24] following incubation with or without AT2220. Conditioned media (15 mL) were collected 2 to 3 days post-transfection, concentrated, and adjusted to pH 6.5. GAA precursors were affinity captured with Concanavalin A-Sepharose beads, washed to remove residual AT2220, and measured for enzymatic activity using the artificial substrate 4-MUG (**Fig. 2A**), and for GAA protein levels by Western blotting (**Fig. 2B**). All four mutants tested showed a marked increase in enzymatic activity, while protein levels were increased maximally by 2-fold (**Table 1**). These data indicate that AT2220 incubation can greatly increase the enzymatic activity of GAA mutants independent of proteolytic processing, and suggest that AT2220-mediated increases in mutant GAA may result from improved folding and/or enhanced stability in the presence of chaperone.

**Table 1:** Effect of AT2220 incubation on enzyme activity and protein levels of secreted wild-type and mutant GAA.

	-Fold Increase		GAA Activity: Protein Ratio
Secreted GAA	*Activity*	*GAA Protein*	
Wild-type	0.90±0.01	0.86±0.19	1.05
P545L	28.06±7.40	2.84±0.52	9.88
M519V	8.00±1.07	1.24±0.06	6.45
G549R	21.88±4.38	1.90±0.16	11.52
L552P	29.46±9.98	1.36±0.18	21.66

COS-7 cells were transfected with wild-type or various mutant forms of GAA, followed by incubation with or without 100 µM AT2220. Increases in the enzymatic activity and total protein levels of secreted GAA were measured as described in ‘Materials and Methods’, and as shown in **Fig. 2**. AT2220-mediated increases in the relative specific activities of the various mutant forms of GAA were calculated as the ratio of GAA enzyme activity to the total GAA protein levels. Activity and protein values represent the mean ± SD from four independent experiments.

### AT2220 promotes the export of P545L GAA from the ER

**Figure 3:**
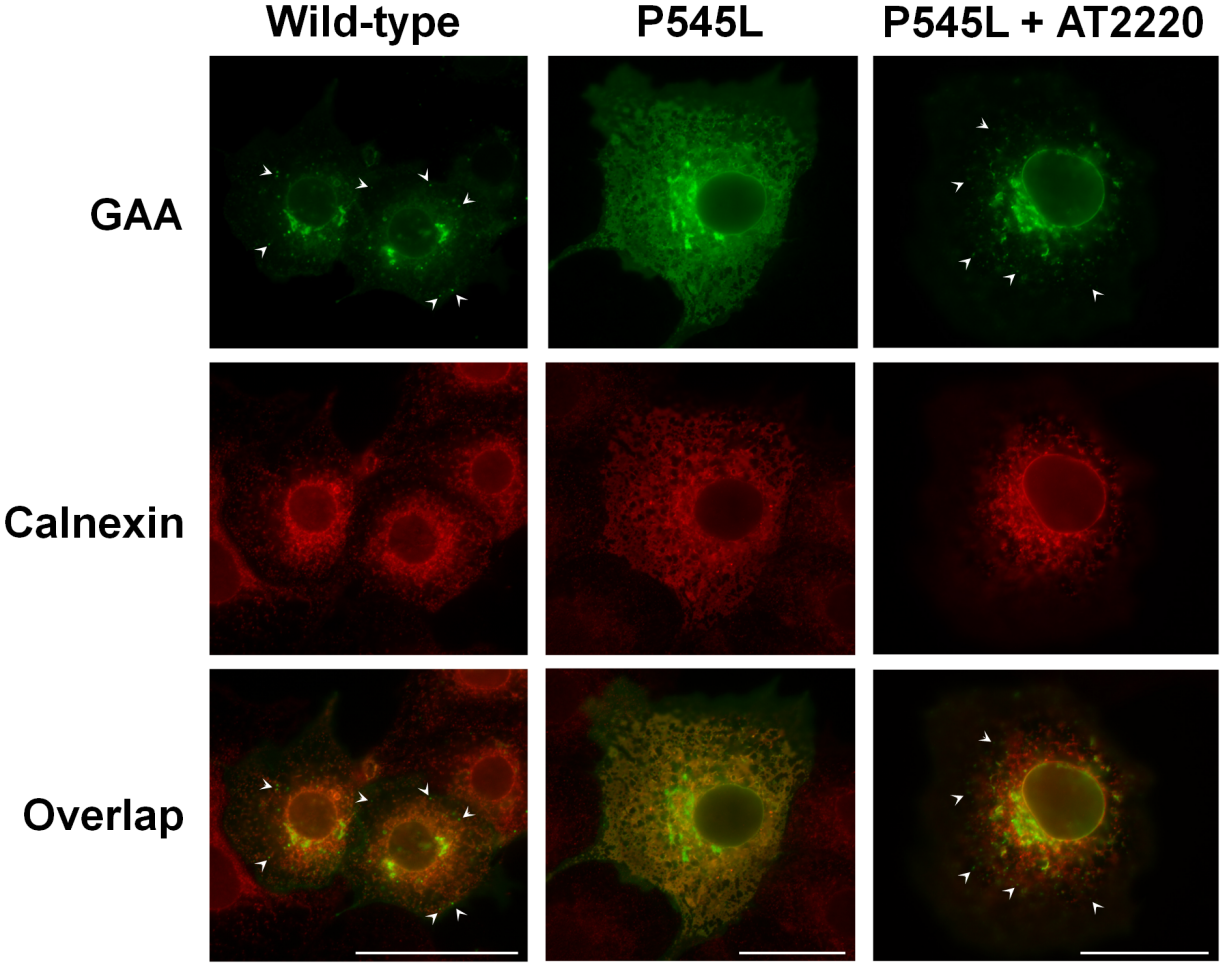
AT2220 promotes the export of P545L GAA from the ER. COS-7 cells were transiently transfected with wild-type or P545L GAA, immediately followed by 3-day incubation with and without 100 µM AT2220. Cells were then fixed and stained with antibodies against GAA (green) and calnexin, a marker for the endoplasmic reticulum (ER; red) as described in ‘Materials and Methods’. Co-localization of the GAA and calnexin signals appears yellow. Puncta (indicative of lysosomal localization) were seen throughout wild-type cells, as well as in P545L cells incubated with AT2220 (arrowheads). Scale bars: 50 µm.

We and others have suggested that AT2220 promotes ER export of some mutant forms of GAA, thus resulting in improved trafficking to lysosomes [24]–[26]. To corroborate the increased levels of the mature, lysosomal forms of hP545L GAA seen when heterologously expressed in COS-7 cells (Fig. 1C) and in patient-derived fibroblasts (Fig. 1E), we examined the ER localization of P545L GAA using epifluorescence microscopy coupled with deconvolution (**Fig. 3**). In the absence of AT2220, P545L GAA immunofluorescence appeared as a diffuse, reticular pattern that co-localized with calnexin, an ER-resident protein. This distribution is in sharp contrast to wild-type GAA, which appeared mainly as puncta distributed throughout the cell (**Fig. 3**, arrowheads). In cells incubated with AT2220, the pattern of P545L GAA immunofluorescence no longer exhibited extensive overlap with calnexin, instead showing a distribution similar to wild-type GAA (**Fig. 3**, arrowheads). In combination with the Western blotting data, these results suggest that P545L GAA is ER-retained in the absence of AT2220, and that incubation with AT2220 promotes its export from the ER.

### AT2220 stabilizes mature P545L GAA in the lysosome

**Figure 4:**
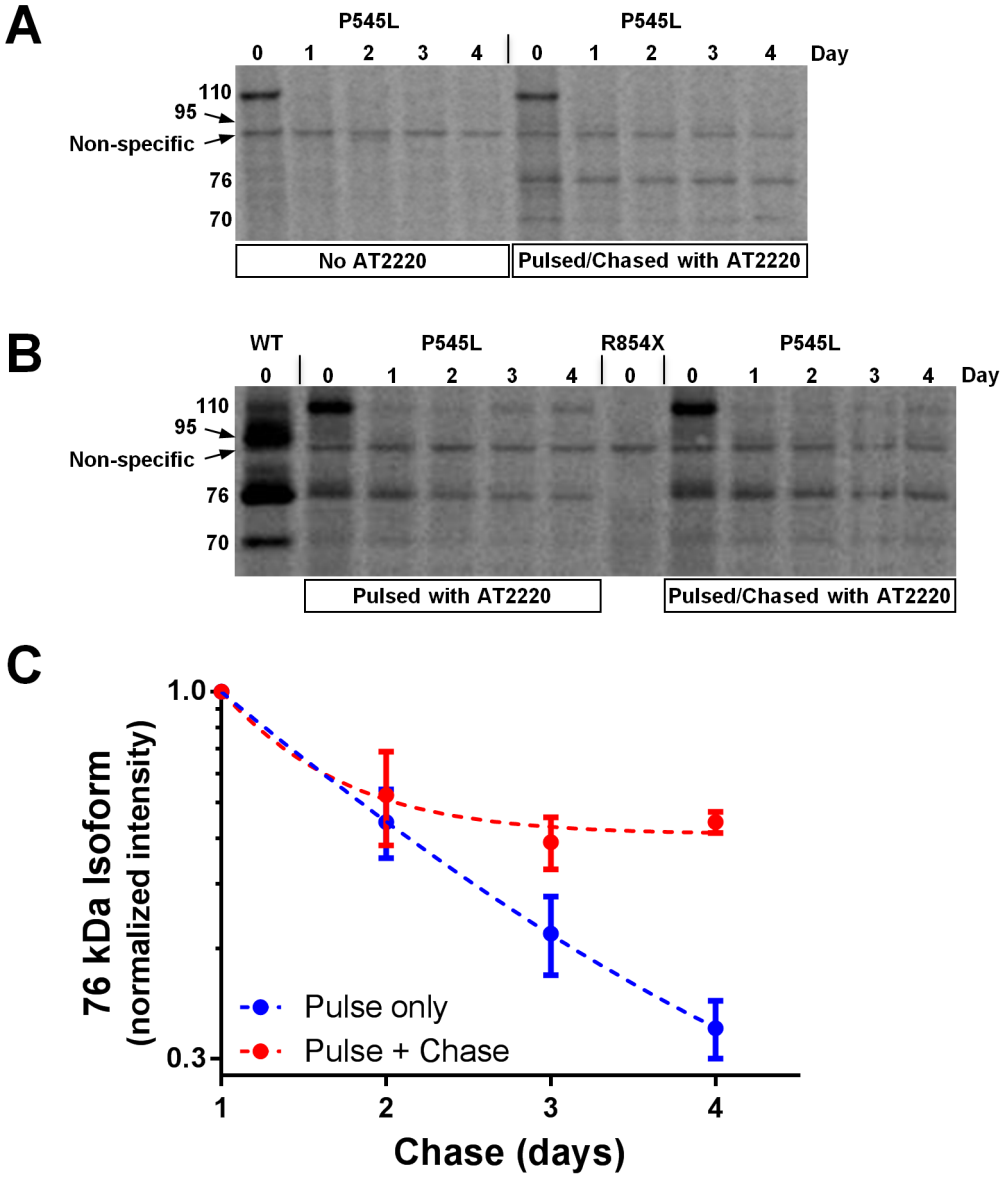
AT2220 increases the stability of mature P545L GAA in lysosomes of Pompe fibroblasts. (**A** and **B**) P545L GAA fibroblasts were incubated with 100 µCi ^35^S-cysteine/methionine for 3 days, chased for 0 to 4 days, then harvested and lysed. Incubation with 100 µM AT2220 during the pulse only, the pulse and the chase, or neither was performed as indicated. Isoforms of GAA (denoted as the 110, 95, 76, and 70 kDa species) were immunoprecipitated from cell lysates, separated by SDS-PAGE, and imaged with a phosphorimager. As controls, immunoprecipitates from metabolically labeled wild-type (denoted as ‘WT’ in the figure) and R854× fibroblasts (both in the absence of AT2220) were also evaluated. (**C**) Levels of the mature 76 kDa isoform of P545L GAA were quantified, normalized against the levels present at the 1-day chase time point, and plotted as a function of time. The data were fitted with an exponential function to estimate the half-life of the 76 kDa isoform of P545L GAA in the presence or absence of AT2220. The 1-day chase time point was chosen as time 0 for the exponential fit in order to eliminate the potential contribution of labeled 110 kDa precursor to the 76 kDa pool.

Pulse-chase experiments were conducted in P545L, wild-type, and R854× fibroblasts incubated with AT2220 during the pulse only, both the pulse and the chase, or neither. In the absence of AT2220, P545L GAA only appeared as the precursor isoform at the beginning of the chase (*i.e.*, on Day 0; **Fig. 4A**), similar to the results shown in **Fig. 1E**. In contrast, the presence of AT2220 during both the pulse and the chase resulted in the appearance of P545L as precursor, intermediate, and mature lysosomal isoforms at the beginning of the chase (Day 0; **Fig. 4A and B**), again similar to **Fig. 1E**, with the mature lysosomal isoforms remaining relatively stable over the 4-day time course (Days 1 through 4; **Fig. 4A–C**). However, when AT2220 was included only in the pulse, the intensity of the mature isoforms of P545L GAA declined over the 4-day time course (**Fig. 4B**), with a half-life of approximately 1.5 days (**Fig. 4C**), indicating that AT2220 can stabilize mature P545L GAA once it has trafficked to, and been processed in, lysosomes. These results suggest that AT2220 is required for P545L GAA trafficking and maturation, and can continue to stabilize the processed mature forms in the lysosome. In wild-type fibroblasts, GAA appeared mainly as the intermediate and mature lysosomal isoforms (**Fig. 4B**), which were stable for at least four days of chase in the absence of AT2220 (data not shown). Again, no GAA protein was seen in Pompe fibroblasts homozygous for the R854× mutation (**Fig. 4B**).

### hP545L GAA Tg/KO mice have reduced GAA activity and elevated glycogen

**Figure 5:**
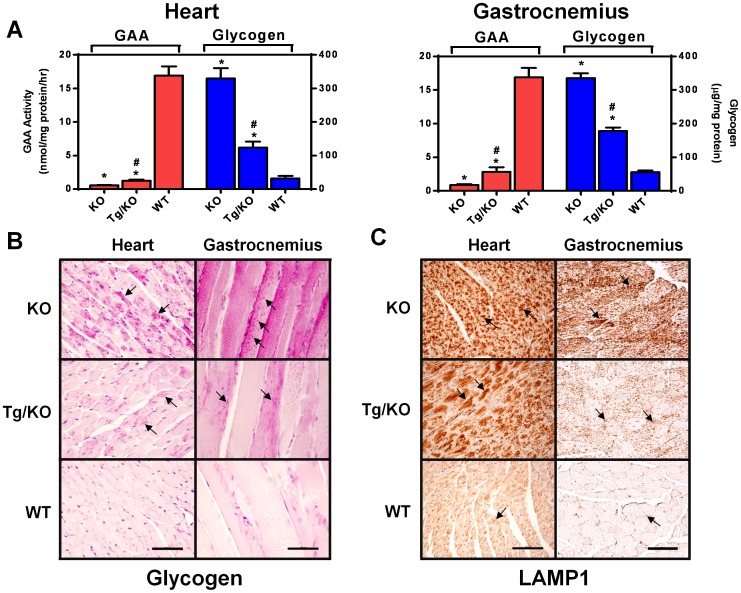
hP545L GAA Tg/KO mice have reduced tissue GAA activity, and elevated glycogen and LAMP1 levels. (**A**) GAA and glycogen levels were assessed in tissue lysates prepared from heart and gastrocnemius of 12-week old male *Gaa* KO, hP545L GAA Tg/KO, and wild-type (denoted as ‘WT’ in the figure) mice. Significant differences in GAA activity and glycogen levels in hP545L GAA Tg/KO and *Gaa* KO mice compared to wild-type mice are reported as *p<0.05, t-test. Significant differences in GAA activity and glycogen levels in hP545L GAA Tg/KO mice compared to *Gaa* KO mice are reported as ^#^p<0.05, t-test. Each bar represents the mean ± SEM from four mice per group analyzed in triplicate. Glycogen accumulation (**B**) and lysosomal proliferation (assessed by the quantity of LAMP1) (**C**) were assessed histochemically in *Gaa* KO, hP545L GAA Tg/KO, and wild-type (denoted as ‘WT’ in the figure) mouse cardiomyocytes and skeletal muscles fibers of the gastrocnemius. Glycogen staining is represented as dark pink spots, and LAMP1 staining as dark brown spots (each denoted with black arrows). The data shown are representative photomicrographs from four mice using a 40× (scale bar: 50 µm) or 20× (scale bar: 100 µm) objective in (**B**) and (**C**), respectively.

Given our *in vitro* findings with P545L GAA, in combination with those previously reported indicating responsiveness to the pharmacological chaperone NB-DNJ, a transgenic mouse model was constructed with this mutant form. Tissue GAA and glycogen levels were measured in 12-week old male hP545L GAA Tg/KO, *Gaa* KO, and age-matched wild-type littermate mice. As representative tissues, the GAA activities in heart and gastrocnemius of hP545L GAA Tg/KO mice were measured and determined to be approximately 10% and 20%, respectively, of those seen in wild-type mouse tissues (**Fig. 5A**). Correspondingly, the glycogen levels in heart and gastrocnemius muscle of hP545L GAA Tg/KO mice were elevated 4- and 3.5-fold, respectively, compared to wild-type mouse tissues, and were approximately 40% and 53%, respectively, of those measured in *Gaa* KO mouse tissues (**Fig. 5A**). Using histochemical methods, a similar glycogen staining pattern was seen in hP545L GAA Tg/KO and *Gaa* KO mice, though the overall signal was higher in *Gaa* KO mice (**Fig. 5B**). Most of the glycogen signal was seen in cardiomyocytes of heart and myocytes/myotubes of gastrocnemius (**Fig. 5B**; dark red spots indicated by black arrows). In addition, hP545L GAA Tg/KO showed lysosomal proliferation as indicated by increased levels of LAMP1 (**Fig. 5C**, dark brown spots indicated by black arrows), similar to observations in *Gaa* KO mice and which is indicative of disease pathology [37]. Collectively, these results indicate that hP545L GAA Tg/KO mice have reduced GAA activity, elevated glycogen levels, and increased lysosomal content in the cells of disease-relevant tissues, including heart and skeletal muscle.

### AT2220 is orally available and broadly distributed in mice

AT2220 (100 mg/kg) was orally available following administration to eight-week old male C57BL/6 mice (**Fig. S1**). At this dose, AT2220 showed a broad biodistribution profile, and attained tissue concentrations that were in excess of its *K_i_* value (approximately 20 nM) for interaction with GAA [26] in both wild-type and hP545L GAA Tg/KO mice (**Fig. S1** and **Table 2**, respectively). At this dose, AT2220 was also accessible to the central nervous system (CNS) in the transgenic mice, with brain concentrations of 1753±110 nM (mean ± SEM; lower limit of quantiation: 184 nM), thus confirming the potential for AT2220 to bind GAA *in vivo* in multiple tissues. A sublinear, dose-dependent increase in the concentration of AT2220 was also seen in disease-relevant tissues of hP545L GAA Tg/KO mice that were orally administered AT2220 (**Table 2**). Four-week administration of 300 mg/kg AT2220 followed by drug withdrawal resulted in a relatively rapid decline in tissue AT2220 levels over the subsequent 7-day period, ultimately falling to levels that were below the limit of quantitation (184 nM) (**Table 2**).

**Table 2:** Tissue levels of AT2220 in hP545L GAA Tg/KO mice following four-week daily oral administration.

*post-AT2220 withdrawal (days)*	AT2220 Tissue Concentrations (nM)							
	Heart				Gastrocnemius			
AT2220 (mg/kg)	*1*	*2*	*5*	*7*	*1*	*2*	*5*	*7*
30	601±55	-	-	-	962±74	-	-	-
100	1226±153	-	-	-	1753±110	-	-	-
300	2243±178	809±221	202±12	LLOQ	3702±221	1973±245	325±31	LLOQ

Eight-week old male hP545L GAA Tg/KO mice were administered AT2220 *ad libitum* in drinking water for four weeks at the indicated doses. Tissues were collected at the indicated times post-AT2220 withdrawal, and AT2220 levels were quantitated using LC-MS/MS as described in ‘Materials and Methods’. AT2220 concentrations are expressed as nM, and represent the mean ± SEM for groups of 7 mice. LLOQ: lower limit of quantitation (184 nM).

### AT2220 increases GAA activity in tissues of hP545L GAA Tg/KO mice

**Figure 6:**
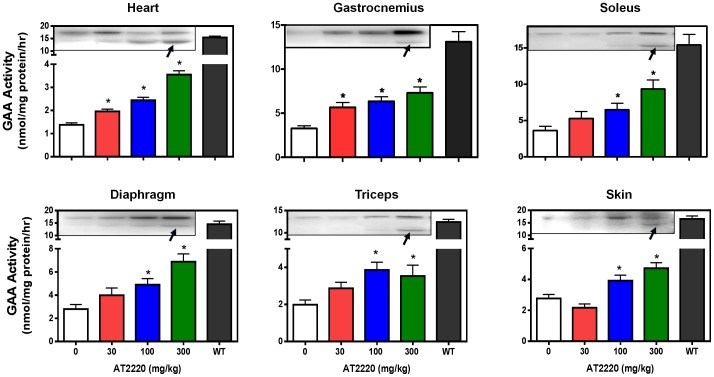
Oral AT2220 administration increases GAA activity in hP545L GAA Tg/KO mouse tissues. Eight-week old male hP545L GAA Tg/KO mice were administered AT2220 *ad libitum* in drinking water for four weeks at the indicated doses. GAA activity was measured in homogenates of heart, gastrocnemius, soleus, diaphragm, triceps, and skin as described in ‘Materials and Methods’. The data presented represent the mean ± SEM of 7 mice/group analyzed in triplicate. Significant increases in GAA activity are reported as *p<0.05 vs. untreated, t-test. The effect was also significant for a linear trend, indicating a dose-dependent increase in GAA activity (post-hoc analysis, p<0.05). **Insets**. Tissue GAA protein levels were determined by Western blotting. The upper band represents the 110 kDa precursor isoform. The appearance of the 76 kDa mature lysosomal form is indicated by the dark arrow. Each lane on each Western blot contains the tissue homogenate from a single animal. The Western blot shown is representative of two experiments, each of which included a different mouse from each group.

AT2220 (30, 100, and 300 mg/kg per day) was administered to eight-week old male hP545L GAA Tg/KO mice *ad libitum* in drinking water for four weeks; AT2220 was then withdrawn, with GAA activity measured 24 hours later. A dose-dependent and significant increase in GAA activity was seen in homogenates from all tissues examined after AT2220 administration (**Fig. 6**). Tissue GAA activity showed maximal increases of 2- to 3-fold and attained 25% to 60% of wild-type littermate levels. Notably, Western blotting revealed that AT2220 administration also results in a dose-dependent increase in total GAA protein in each tissue, and importantly the appearance of the 76 kDa mature lysosomal form of GAA (**Fig. 6** **, insets**; indicated by black arrows). Collectively, these data are consistent with the observations *in vitro*, and suggest that AT2220 can also stabilize mutant P545L GAA *in vivo*, thereby increasing overall protein levels, processing, and lysosomal delivery in the cells of various disease-relevant tissues.

### AT2220 administration reduces glycogen levels in hP545L GAA Tg/KO mice

**Figure 7:**
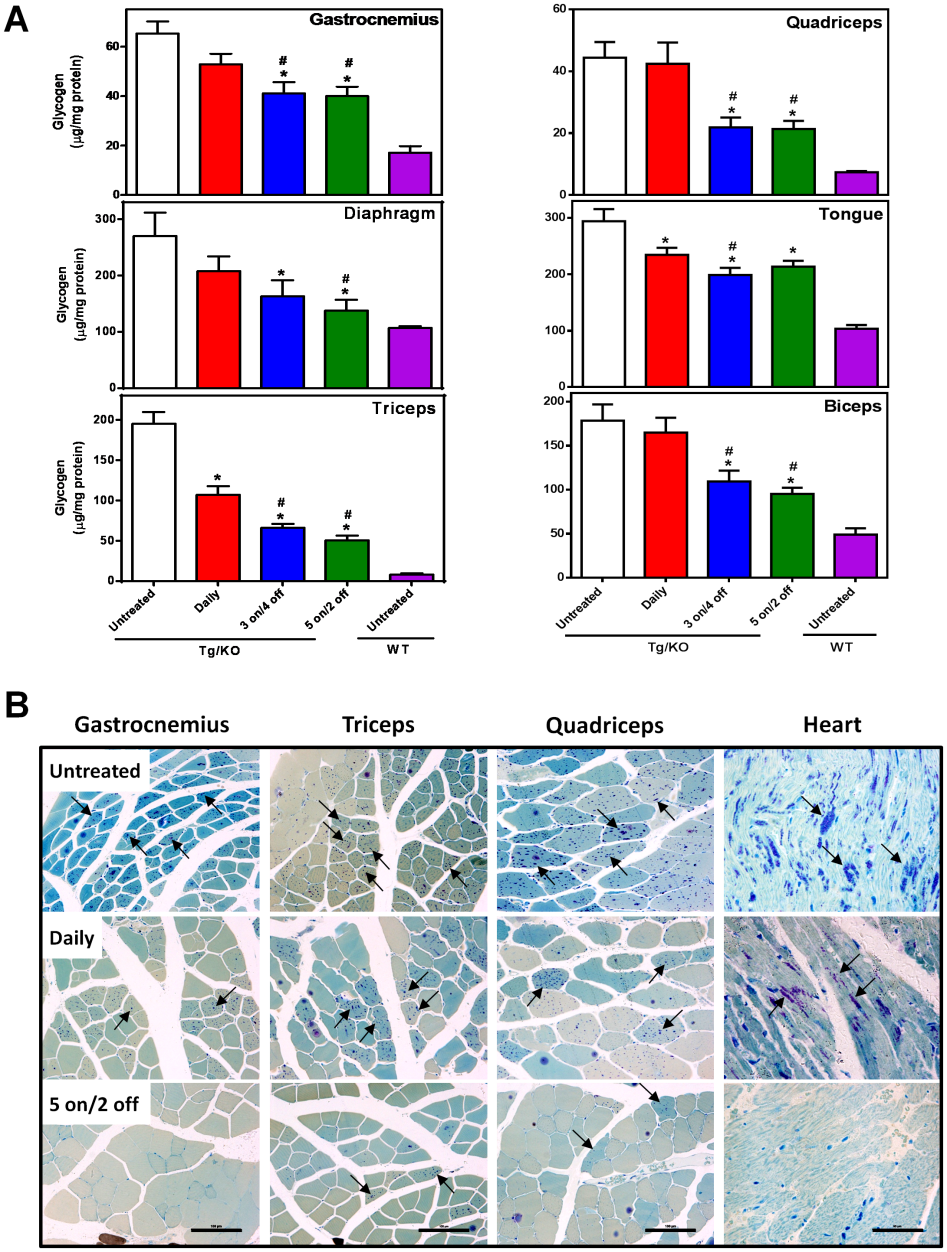
Oral AT2220 administration reduces tissue glycogen levels in hP545L GAA Tg/KO mice. (**A**) Eight-week old male hP545L GAA Tg/KO mice were administered 100 mg/kg per day AT2220 *ad libitum* in drinking water for four weeks either daily or less-frequently using four cycles of a ‘3 on/4 off’ or a ‘5 on/2 off’ regimen. Mice were euthanized 24 hours after AT2220 withdrawal in the daily regimen, and after 4 or 2 days off in the ‘3 on/4 off’ and ‘5 on/2 off’ regimens, respectively. The data presented represent the mean ± SEM of 7 mice/group analyzed in triplicate. Significant reductions in glycogen levels are reported as *p<0.05 vs. untreated, t-test, or ^#^p<0.05 daily vs. less-frequent, t-test. WT, wild-type. (**B**) Cell type-specific reduction of glycogen in gastrocnemius, triceps, quadriceps, and heart of hP545L GAA Tg/KO mice administered 100 mg/kg/day AT2220 *ad libitum* in drinking water either daily or less-frequently (5 on/2 off’). The glycogen signal is represented as dark blue spots denoted with black arrows. The data shown are representative photomicrographs from 7 mice/group (magnification: 20X). Scale bars: 100 µm for gastrocnemius, triceps, and quadriceps; 50 µm for heart.

Eight-week old male hP545L GAA Tg/KO mice were administered AT2220 (100 mg/kg per day) *ad libitum* in drinking water either daily or less-frequently using a ‘3 on/4 off’ (three consecutive days with AT2220 followed by four consecutive days with drinking water only) or a ‘5 on/2 off’ (five consecutive days with AT2220 followed by two consecutive days with drinking water only) regimen for four weeks. Mice were euthanized 24 hours after AT2220 withdrawal in the daily regimen, and after 4 or 2 days off in the ‘3 on/4 off’ and ‘5 on/2 off’ regimens, respectively. Daily administration of AT2220 significantly reduced glycogen levels in some tissues of hP545L GAA Tg/KO mice (**Fig. 7A**). However, in all tissues examined, there was a significantly greater reduction in glycogen levels with the less-frequent administration regimens compared to daily administration (**Fig. 7A** and **Table 3**). Notably in heart, complete glycogen clearance was seen following either of the less-frequent AT2220 administration regimens; that is, cardiac glycogen levels were similar to those seen in wild-type littermates (**Table 3**). Histological results also revealed greater glycogen reduction in myocytes/myotubes of heart and skeletal muscles with less-frequent AT2220 administration compared to daily (**Fig. 7B**). Interestingly, histological measurements in heart and soleus also revealed reduced lysosomal proliferation as indicated by a lower LAMP1 signal (**Fig. S2**). AT2220 also reduced glycogen levels in brain and spinal cord of hP545L GAA Tg/KO mice, with significantly greater reduction seen with the less-frequent regimens compared to daily administration (**Table 3** and **Fig. S3**).

**Table 3:** Effect of four-week daily or less-frequent AT2220 administration on tissue glycogen levels in hP545L GAA Tg/KO mice.

Tissue	Glycogen (% Reduction)		
	*Daily*	*3 on/4 off*	*5 on/2 off*
Gastrocnemius	−26±9	−50±9*^#^	−53±8*^#^
Quadriceps	−5±18	−61±8*^#^	−62±7*^#^
Diaphragm	−38±16	−66±17*	−81±12*^#^
Tongue	−31±7*	−50±7*^#^	−42±5*
Triceps	−47±6*	−69±3*^#^	−77±3*^#^
Biceps	−10±13	−56±10*^#^	−68±6*^#^
Heart	−37±17*	−104±6*^#^	−101±6*^#^
Soleus	−36±8	−56±10*^#^	−85±6*^#^
Brain	−33±13	−61±10*^#^	−50±11*
Spinal cord	−35±10	−82±7*^#^	−63±7*

Eight-week old male hP545L GAA Tg/KO mice were administered 100 mg/kg AT2220 *ad libitum* in drinking water for four weeks using the indicated regimens. Tissue glycogen levels were determined as described in “Materials and Methods”. The glycogen levels were normalized relative to baseline levels of untreated hP545L GAA Tg/KO (100%) and wild-type mice (0%). *p<0.05 compared to baseline; #p<0.05 compared to daily, as determined by t-test. Values represent the mean ± SEM for 7 mice per group.

## Discussion

Previous *in vitro* studies have shown that the pharmacological chaperone AT2220 (1-deoxynojirimycin hydrochloride; duvoglustat hydrochloride) directly binds and stabilizes wild-type GAA, and increases the stability, trafficking, and maturation of different mutant forms of GAA in transfected cells and Pompe patient-derived fibroblasts, thereby leading to increased cellular levels of GAA activity [24]–[26]. However, the precise mechanism of action of AT2220 on mutant GAA remains unclear. Here we show that AT2220 has multiple modes of action during GAA synthesis and maturation that ultimately lead to increased delivery of active GAA to the lysosome and greater reduction of its natural substrate, glycogen, *in vivo*.

In our cell-based studies, a clear increase in the specific activity of AT2220-responsive mutant forms of GAA was observed using the artificial substrate 4-MUG, as well as glycogen. Furthermore, AT2220 improved the catalytic activity of the precursor forms of multiple GAA mutants *prior* to proteolytic processing into its mature lysosomal forms. While increases in glycogen hydrolysis have been attributed to increased levels of mature GAA [20], [22], our studies indicate that the synthesis of precursor forms in the presence of AT2220 can result in catalytic improvement against 4-MUG. The AT2220-mediated improvements in mutant GAA activity may be due to changes in *de novo* folding that result in more efficient substrate turnover. A similar effect was noted with isofagomine on mutant glucocerebrosidase (GCase) in Gaucher patient-derived fibroblasts [38]. While isofagomine increased the specific activity of N370S GCase by approximately 30%, the effect of AT2220 on the specific activity of mutant GAA was much more pronounced, with increases ranging from 50% to 600% depending on the mutant form.

AT2220 also promotes the trafficking of multiple mutant forms of GAA, permitting exit from the ER, passage through the secretory pathway, and delivery to lysosomes where processing to the mature 76 and 70 kDa forms occurs. Together these results suggest that AT2220 improves the folding of mutant GAA, resulting in increased catalytic activity, passage through the ER quality control, and enhanced stability in lysosomes. In fact, a recent survey of Pompe disease-related mutant forms of GAA revealed a strong correlation between residual enzyme activity and trafficking to lysosomes as evident through proteolytic processing [26]. Over 80% of the GAA mutants tested that had less than 2% of wild-type activity showed no indication of lysosomal trafficking, while over 70% of mutants tested that had greater that 2% of wild-type activity did show lysosomal trafficking. These findings suggest that the AT2220-induced conformational changes that promote mutant GAA trafficking may also enhance its catalytic activity.

Our studies also indicate that AT2220 increases the stability of mutant GAA precursors that are secreted into the culture media, as well as the mature GAA isoforms in lysosomes. For the GAA precursors that were secreted from transfected COS-7 cells, AT2220 increased the specific activity nearly ten-fold. This large change in specific activity results from more than just increased catalytic activity due to improved folding, as it was substantially greater than the changes seen for the intracellular forms of each respective mutant. Our previous study demonstrated that AT2220 confers increased thermal stability to wild-type GAA that is time-, temperature-, and pH-dependent, with low stability seen at neutral pH [26], [35]. Taken together, these results suggest that AT2220 stabilizes mutant GAA precursors that are secreted into the cell culture medium, protecting them from denaturation. In addition, AT2220 markedly increased the cellular half-life of the mature lysosomal forms of mutant GAA, suggesting a chaperone-mediated protection against degradation by lysosomal proteases, and indicating an additional mode of action even after maturation of the enzyme. Stabilization of mutant protein by chaperone in the lysosome may allow for a buildup of enzyme levels in the target organelle that are sufficient to reduce glycogen after the chaperone has been cleared. The studies in hP545L Tg/KO mice support this hypothesis.

The *in vivo* effects of AT2220 on P545L GAA were investigated using a newly generated mouse model of Pompe disease. This model expresses a human transgene of mutant P545L GAA on a *Gaa* knock-out background (hP545L GAA Tg/KO), and shows low tissue GAA activity and elevated glycogen levels in disease-relevant tissues including heart, diaphragm, multiple skeletal muscles, and brain. While assessments of muscle function and strength have not been made in these mice and represent key areas of future experimentation, the hP545L GAA Tg/KO mice do represent an excellent biochemical model of Pompe disease. To this end, daily oral administration of AT2220 for four weeks resulted in dose-dependent and significant increases in GAA activity in disease-relevant tissues. As also seen in our cell-based studies, AT2220 increased the levels of the 110 kDa precursor form of P545L GAA, and led to the appearance of the mature 76 kDa lysosomal form, in these tissues. Importantly, these increased levels of mature, lysosomal GAA translated into greater tissue glycogen reduction in mice administered AT2220 daily compared to vehicle administration.

The reduction of tissue glycogen levels in hP545L GAA Tg/KO mice was further optimized using regimens that administered AT2220 less frequently. This was based on the hypothesis that the shorter tissue half-life of AT2220 would allow for a more rapid clearance compared to the longer half-life of elevated, lysosomal GAA (multiple days) [39], and that this difference could be exploited to produce a larger net gain in lysosomal enzyme activity while administering less total drug [32]. Specifically, 3- or 5-day administration of AT2220 could provide a period of enhanced protein stabilization and trafficking to lysosomes (maximal chaperone effect), followed by a 4- or 2-day withdrawal of AT2220, respectively, to allow for dissociation and tissue efflux of the chaperone, thus maximizing *in situ* GAA activity. Indeed, greater glycogen reduction was realized in all tissues using the less-frequent regimens compared to daily administration. In heart, the glycogen levels reached wild-type levels, similar to what was previously reported following administration of 20 mg/kg rhGAA alone [40]. And in skeletal muscles, less-frequent AT2220 administration resulted in glycogen reductions that were similar to those reported previously with 40 mg/kg rhGAA alone [40].

Nearly all of the muscle types evaluated in our study are predominantly type 2 fast twitch fibers, which encompass at least 80% of the total fiber type in the muscles we evaluated, with some comprised of over 90% type 2 fibers [41]. In contrast, we also evaluated soleus, which is comprised of an approximately 1∶1 ratio of type 1 and 2 fibers. Based on our glycogen data, soleus showed a greater reduction in glycogen levels, especially in the less-frequent ‘5 on/2 off’ regimen (85% reduction) compared to those seen in other type 2-dominant skeletal muscles such as gastrocnemius (53% reduction), quadriceps (62% reduction), triceps (77% reduction), and biceps (68% reduction). These data suggest that glycogen clearance from slow twitch muscle fibers may be more efficient compared to fast twitch fibers, an observation similar to that reported with ERT [41].

Recent studies indicate that there is also a significant neuropathological component associated with Pompe disease that is characterized by profound pathology largely in the motoneurons of both patients and *Gaa* KO mice, potentially due to widespread glycogen deposition in the peripheral and central nervous system (CNS) [42]–[44]. Importantly, AT2220 was found to cross the blood-brain barrier in mice, with less-frequent AT2220 administration significantly reducing brain glycogen levels. In contrast, multiple administrations of rhGAA had no effect on brain GAA activity or glycogen levels in preclinical or clinical studies [14], [45], underscoring the challenges associated with CNS penetration of exogenous replacement enzymes. These data suggest that AT2220 may offer an advantage over ERT for treating the CNS manifestations of Pompe disease, which should be explored further.

We have shown previously that only a subset of GAA mutants respond to AT2220 [26]. Given that most GAA missense mutations are rare, or ‘private’, and that most late-onset patients have at least one copy of a common splicing mutation [46], the fraction of Pompe patients who have responsive mutant *missense* forms is unknown, though it is expected to be low (our unpublished estimates suggest ∼15%). To this end, the P545L variant has only been identified in a small number of Pompe patients [47]. However, there is evidence that certain GAA mutations are more common in certain geographical regions or within certain ethnic groups [1], [48], some of which may be enriched for chaperone-responsive mutant forms [49], [50]. Given this potentially low prevalence of responsive mutant forms, AT2220 has also recently been investigated in co-administration studies with rhGAA [35], [51]. We and others have shown that AT2220 binds and stabilizes exogenous rhGAA, thereby leading to improved cellular uptake [35], [51] and glycogen reduction [35] in disease-relevant tissues, including skeletal muscles.

Notably, repeat administration of rhGAA to *Gaa* KO mice is also known to result in an immune response that manifests as severe anaphylaxis, often leading to death. The *Gaa* KO mice form anti-rhGAA IgGs that not only limit the number of injections of rhGAA that can be administered before the onset of anaphylaxis, but may also impact efficacy [14]. These observations are similar to what has been reported in some Pompe patients, where rhGAA infusion leads to severe immune responses that are mediated by IgGs, some of which inhibit the catalytic activity of GAA (*i.e*., are neutralizing) [17]. Due to the severity of the immune response in *Gaa* KO mice, efficacy studies are typically limited to a maximum of four rhGAA administrations. Importantly however, the hP545L GAA Tg/KO mice are able to tolerate multiple rhGAA injections (up to 8 in our studies; data not shown), and generate significantly lower IgG levels compared to those seen in *Gaa* KO mice (**Fig. S4**). These observations are most likely due to expression of the mutant human P545L protein in the transgenic mice, which may mimic the cross-reactive immunologic material (CRIM) seen in CRIM-positive Pompe patients. Importantly, these patients typically show substantially better efficacy and tolerability to ERT in the clinic [16]–[17] compared to patients that do not produce GAA (referred to as CRIM-negative) [17]. Collectively, these data suggest that the newly developed hP545L GAA Tg/KO mouse model may serve as a useful tool for further preclinical investigations, including long-term rhGAA administration studies, potentially in combination with pharmacological chaperones, as well as novel substrate reduction therapies.

Taken together, our data indicate that the mechanism by which AT2220 increases the activity of several mutant forms of GAA involves improved catalytic function and ER export, enhanced lysosomal delivery and maturation, as well as protection against degradation in lysosomes. Furthermore, oral administration of AT2220 increases GAA activity, and significantly decreases accumulated glycogen, in disease-relevant tissues of hP545L GAA Tg/KO mice, including heart, diaphragm, skeletal muscles, and brain. Importantly, dose optimization studies revealed that even greater glycogen reduction could be achieved using less-frequent AT2220 administration regimens that result in a larger net gain in lysosomal activity with reduced overall drug exposure. These data suggest that further evaluation of AT2220 as a therapeutic for Pompe patients who have responsive mutant forms of GAA [26] may be warranted.

## Supporting Information

Figure S1 **Eight-week old male wild-type C57BL/6 mice were orally administered 100 mg/kg AT2220.** Plasma, heart, quadriceps, gastrocnemius, and triceps were collected 0, 0.5, 2, 4, 24, 48, and 72 hours post-administration. AT2220 levels were quantitated using LC-MS/MS as described in ‘Materials and Methods’ of the main paper. AT2220 was orally available, and showed a broad tissue distribution profile, attaining tissue concentrations that were in excess of its *K_i_* value (approximately 20 nM) for interaction with GAA. Each time point represents the mean ± SEM of 3 mice/group.
(TIF)

Figure S2 **Lysosomal proliferation occurs in hP545L GAA Tg/KO mice.** Tissue LAMP1 levels were measured in 12-week old male hP545L GAA Tg/KO and age-matched littermate wild-type mice as described in “Materials and Methods” of the main paper. Immunohistochemistry using LAMP1 staining revealed reduced lysosomal proliferation in cardiomyocytes of heart and myocytes/myotubes of soleus following AT2220 administration. LAMP1 staining is represented as dark brown spots, denoted with black arrows. The data shown are representative photomicrographs from 7–8 mice/group (magnification: 20X). Scale bars: 50 µm for heart; 100 µm for soleus.
(TIF)

Figure S3 **AT2220 reduces glycogen levels in the CNS of hP545L GAA Tg/KO mice.** Twelve-week old male hP545L GAA Tg/KO mice were administered 100 mg/kg AT2220 *ad libitum* in drinking water for four weeks either daily or less-frequently. (**A**) Glycogen levels in brain and spinal cord were measured 24 hours after AT2220 withdrawal in the daily group, and 4 or 2 days after withdrawal in the ‘3 on/4 off’ and ‘5 on/2 off’ groups, respectively, as described in ‘Materials and Methods’ of the main paper. Significantly greater glycogen reductions were seen with the less-frequent regimens compared to daily administration (*p<0.05 vs. untreated, t-test, ^#^p<0.05 daily vs. less-frequent, t-test). Each bar represents the mean ± SEM of 7–8 mice/group analyzed in triplicate. Twenty-four hours after drug withdrawal, GAA activity levels in brain and spinal cord were each increased approximately 1.5-fold following daily AT2220 administration (data not shown). (**B**) Cell type-specific reduction of glycogen in brain (cortex) and spinal cord of hP545L GAA Tg/KO mice was assessed by immunohistochemistry as described in ‘Materials and Methods’ of the main paper. Glycogen staining is represented as dark pink spots denoted with black arrows. Glycogen content was assessed by the amount and intensity of the signal, and showed a greater reduction with the less-frequent regimen (‘3 on/4 off’) compared to daily administration. The data shown are representative photomicrographs from 7 mice/group (magnification: 20X). Scale bars: 100 µm for brain; 500 µm for spinal cord.
(TIF)

Figure S4 **Repeat administration of rhGAA to hP545L GAA Tg/KO mice does not lead to high IgG levels.** Twelve-week old male hP545L GAA Tg/KO or *Gaa* KO mice were administered rhGAA (20 mg/kg) via bolus tail vein injection every week for 8 weeks (8 total injections), or every other week for 8 weeks (4 total injections), respectively. Plasma was collected 14 days following the last administration (transgenic mice), or 7 and 21 days following the last administration (*Gaa* KO mice), and IgG titers were determined. Briefly, Immulon 2 HB plates (Thermo Fisher Scientific, Waltham, MA) were coated with 5 µg/mL rhGAA in PBS using 100 µL/well, and incubated overnight at 4°C. Each well was then washed three times with 250 µL 0.1% Tween-20 in PBS to remove unbound rhGAA. Plates were blocked for 1 hour at room temperature using 150 µL/well 5% non-fat milk, 0.1% Tween-20 in PBS. Plates were then washed three times as described above, followed by the addition of 100 µL/well of serially diluted plasma samples (range 1∶100 to 1∶51000). Plates were incubated at 37°C for 1 hour, then washed as described above, followed by 1-hour incubation at room temperature with 100 µL/well of 1∶5000 diluted horseradish peroxidase-conjugated donkey anti-mouse IgG (ThermoPierce, Jackson Immunosearch Labs, West Grove, PA). Unbound secondary antibody was washed away, and 100 µL/well Turbo TMB ELISA substrate (Thermo Fisher Scientific) was added and incubated at room temperature for 5 to 10 minutes for color development. The reaction was stopped by the addition of 50 µL/well 1 M H_2_SO_4_ and absorbance was read at 450 nm on a Victor^3^ plate reader (Perkin Elmer, Waltham, MA). To determine IgG titers, an arbitrary cutoff value was set as 2-times the Abs_450 nm_ level measured for blanks (defined as the value produced with Lysis Buffer only). The titer of each plasma sample was recorded as the last dilution factor with an Abs_450 nm_ value that was greater than the cutoff value. Values represent the mean of 4 to 7 mice/group. Titers in the *Gaa* KO mice were comparable to one another, and significantly higher than the titers measured in the hP545L GAA Tg/KO mice. Overall, these data indicate that despite the lower total number of rhGAA injections (four) given to the *Gaa* KO mice, the IgG titers were significantly higher than those seen in hP545L GAA Tg/KO that were administered a greater number (eight) of rhGAA injections.
(TIF)
