# Supplementary figures and images for: The Pharmacological Chaperone AT2220 Increases the Specific Activity and Lysosomal Delivery of Mutant Acid Alpha-Glucosidase, and Promotes Glycogen Reduction in a Transgenic Mouse Model of Pompe Disease
Source: PLoS One. 2014 Jul 18;9(7):e102092. doi: 10.1371/journal.pone.0102092 (PMC4103853; doi:10.1371/journal.pone.0102092)

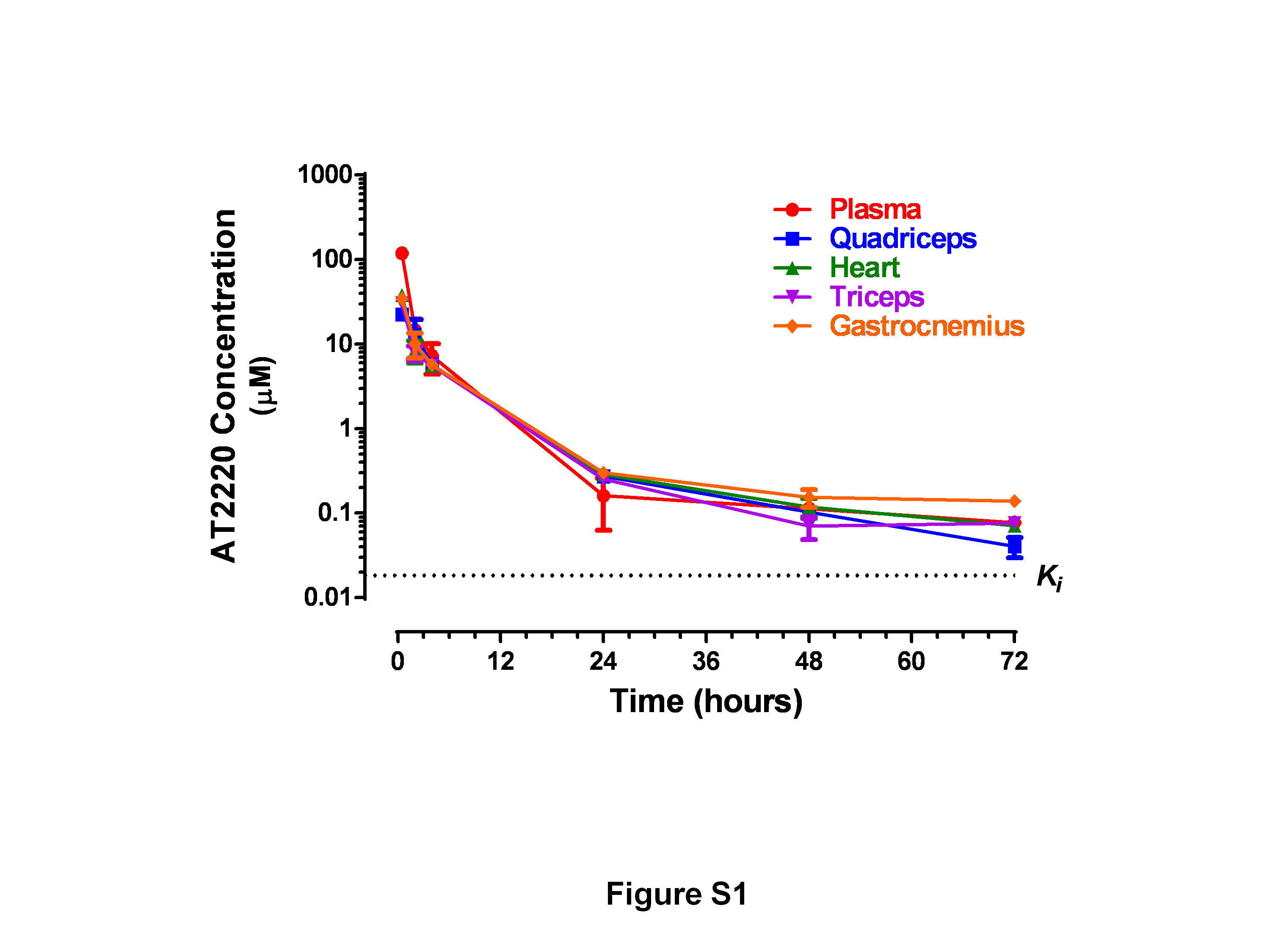

Supplement: Figure S1 — Eight-week old male wild-type C57BL/6 mice were orally administered 100 mg/kg AT2220. Plasma, heart, quadriceps, gastrocnemius, and triceps were collected 0, 0.5, 2, 4, 24, 48, and 72 hours post-administration. AT2220 levels were quantitated using LC-MS/MS as described in ‘Materials and Methods’ of the main paper. AT2220 was orally available, and showed a broad tissue distribution profile, attaining tissue concentrations that were in excess of its Ki value (approximately 20 nM) for interaction with GAA. Each time point represents the mean ± SEM of 3 mice/group. (TIF) [file pone.0102092.s001.tif]

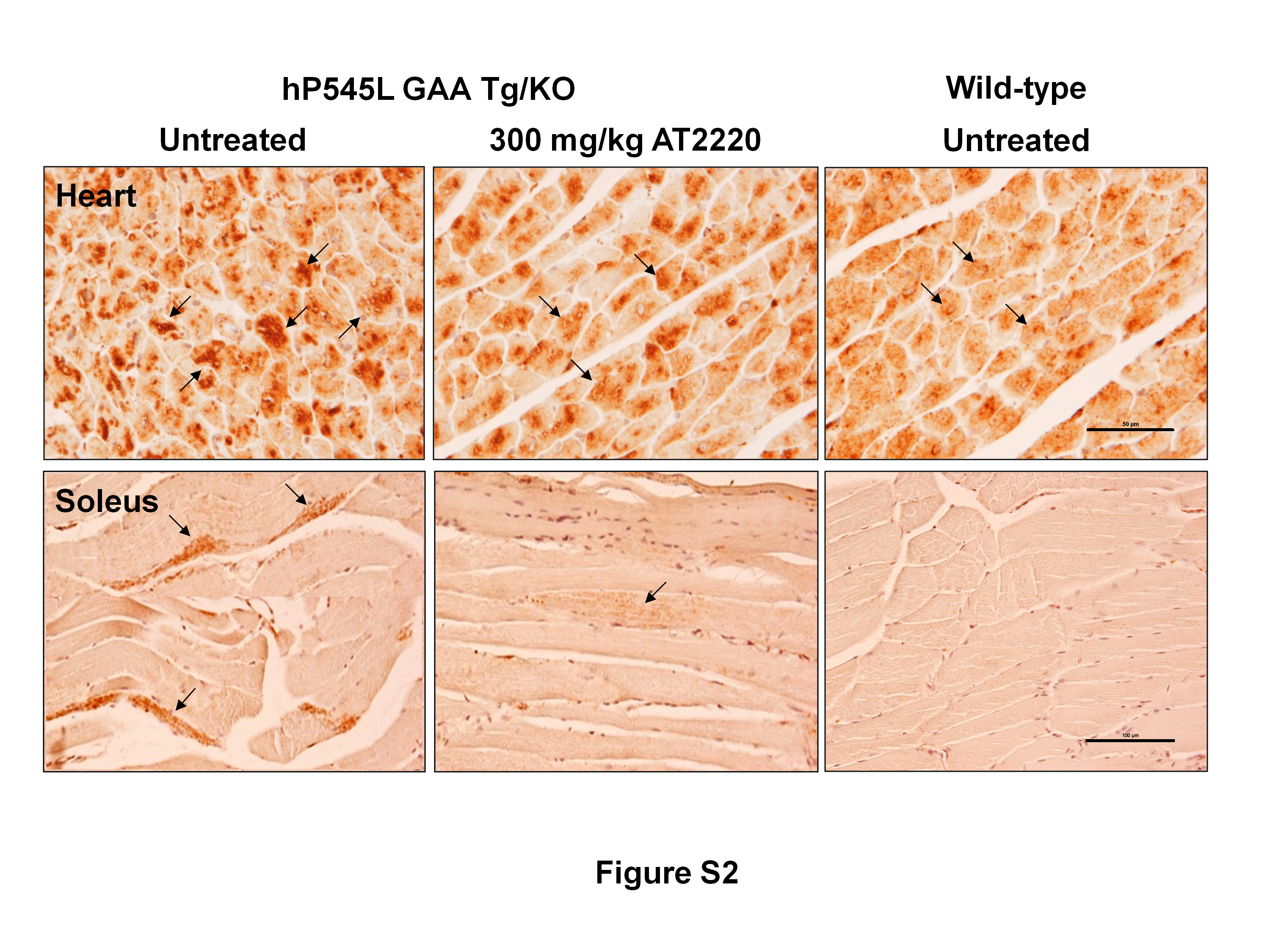

Supplement: Figure S2 — Lysosomal proliferation occurs in hP545L GAA Tg/KO mice. Tissue LAMP1 levels were measured in 12-week old male hP545L GAA Tg/KO and age-matched littermate wild-type mice as described in “Materials and Methods” of the main paper. Immunohistochemistry using LAMP1 staining revealed reduced lysosomal proliferation in cardiomyocytes of heart and myocytes/myotubes of soleus following AT2220 administration. LAMP1 staining is represented as dark brown spots, denoted with black arrows. The data shown are representative photomicrographs from 7–8 mice/group (magnification: 20X). Scale bars: 50 µm for heart; 100 µm for soleus. (TIF) [file pone.0102092.s002.tif]

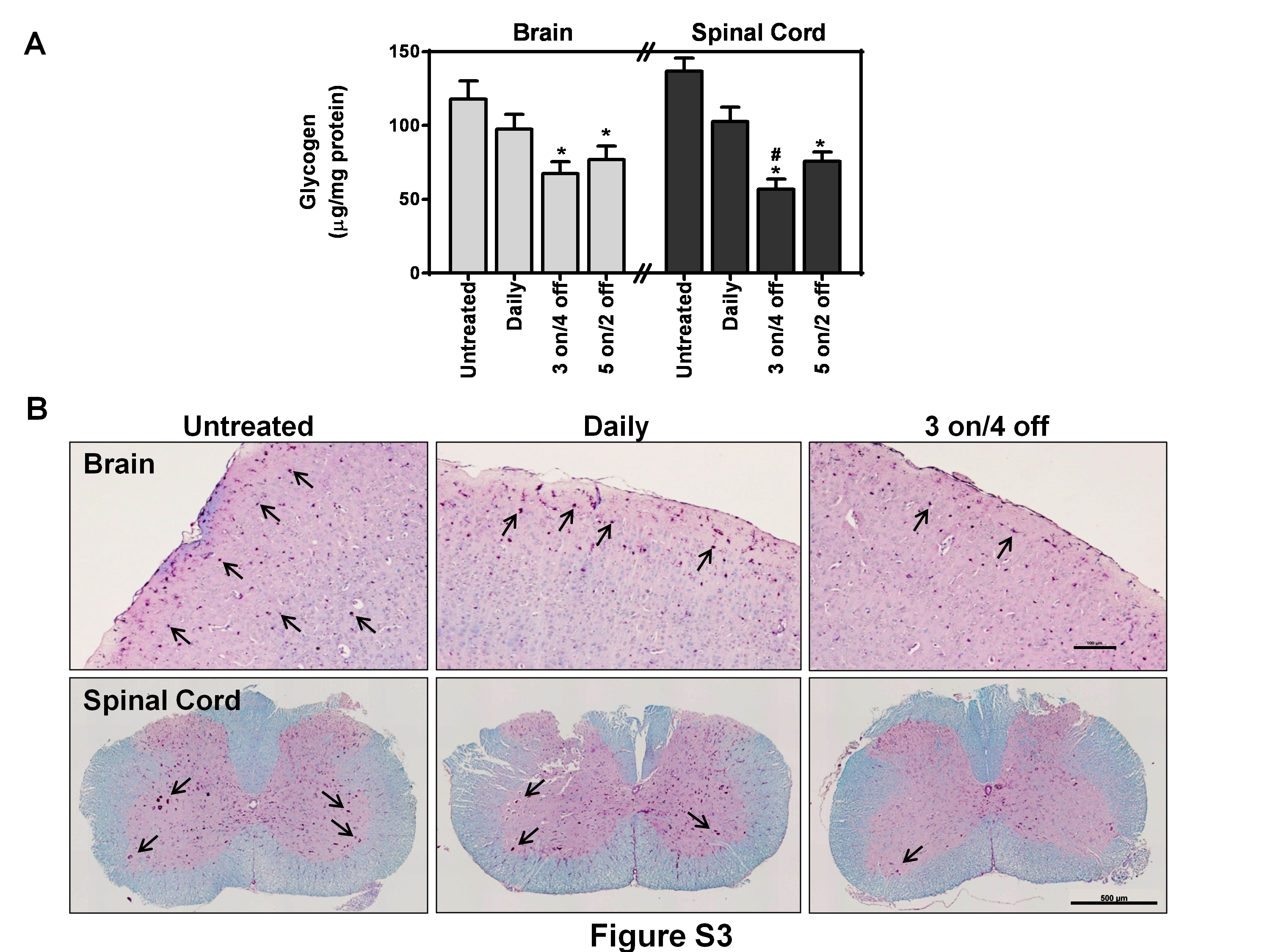

Supplement: Figure S3 — AT2220 reduces glycogen levels in the CNS of hP545L GAA Tg/KO mice. Twelve-week old male hP545L GAA Tg/KO mice were administered 100 mg/kg AT2220 ad libitum in drinking water for four weeks either daily or less-frequently. (A) Glycogen levels in brain and spinal cord were measured 24 hours after AT2220 withdrawal in the daily group, and 4 or 2 days after withdrawal in the ‘3 on/4 off’ and ‘5 on/2 off’ groups, respectively, as described in ‘Materials and Methods’ of the main paper. Significantly greater glycogen reductions were seen with the less-frequent regimens compared to daily administration (*p<0.05 vs. untreated, t-test, #p<0.05 daily vs. less-frequent, t-test). Each bar represents the mean ± SEM of 7–8 mice/group analyzed in triplicate. Twenty-four hours after drug withdrawal, GAA activity levels in brain and spinal cord were each increased approximately 1.5-fold following daily AT2220 administration (data not shown). (B) Cell type-specific reduction of glycogen in brain (cortex) and spinal cord of hP545L GAA Tg/KO mice was assessed by immunohistochemistry as described in ‘Materials and Methods’ of the main paper. Glycogen staining is represented as dark pink spots denoted with black arrows. Glycogen content was assessed by the amount and intensity of the signal, and showed a greater reduction with the less-frequent regimen (‘3 on/4 off’) compared to daily administration. The data shown are representative photomicrographs from 7 mice/group (magnification: 20X). Scale bars: 100 µm for brain; 500 µm for spinal cord. (TIF) [file pone.0102092.s003.tif]

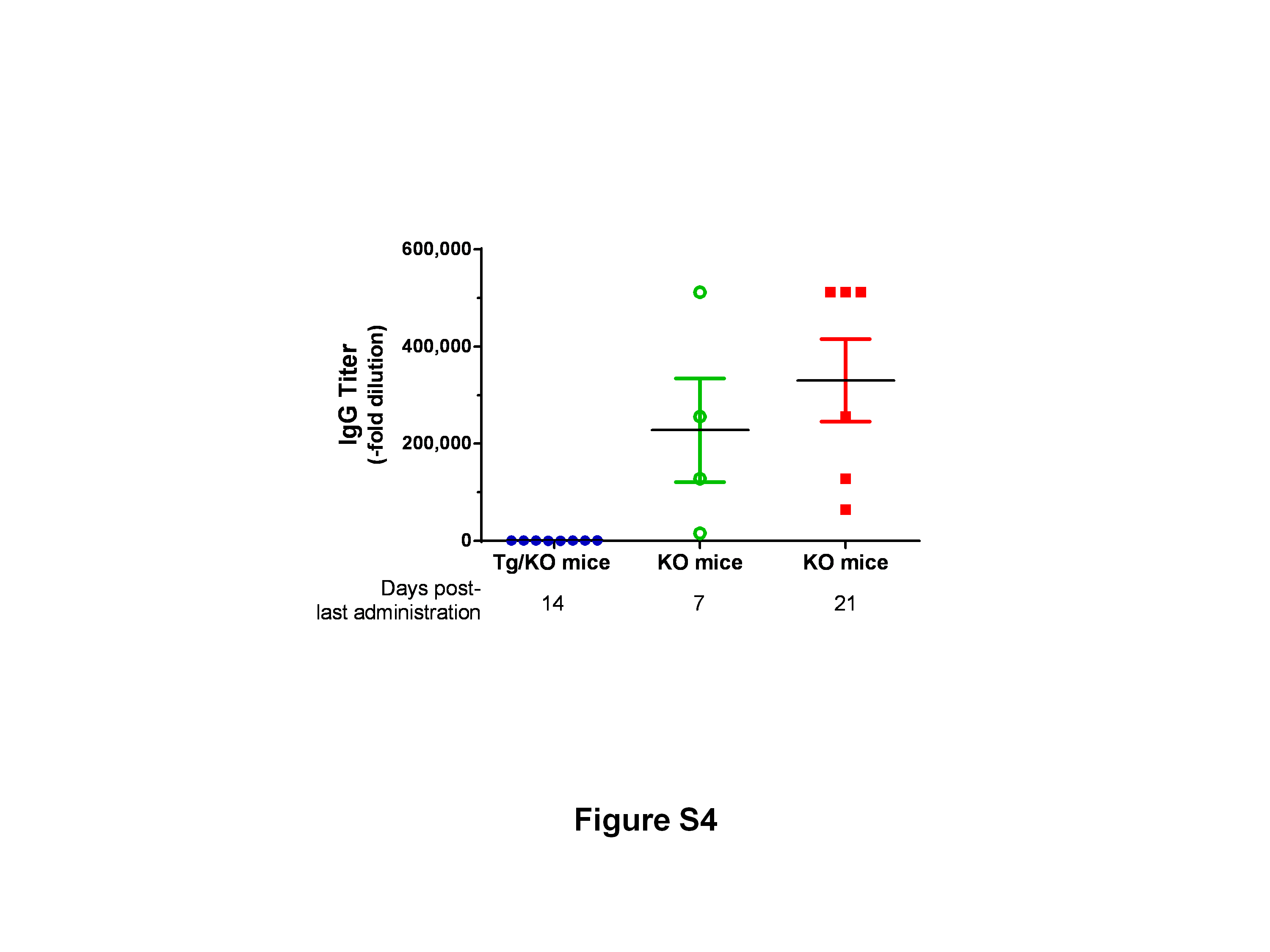

Supplement: Figure S4 — Repeat administration of rhGAA to hP545L GAA Tg/KO mice does not lead to high IgG levels. Twelve-week old male hP545L GAA Tg/KO or Gaa KO mice were administered rhGAA (20 mg/kg) via bolus tail vein injection every week for 8 weeks (8 total injections), or every other week for 8 weeks (4 total injections), respectively. Plasma was collected 14 days following the last administration (transgenic mice), or 7 and 21 days following the last administration (Gaa KO mice), and IgG titers were determined. Briefly, Immulon 2 HB plates (Thermo Fisher Scientific, Waltham, MA) were coated with 5 µg/mL rhGAA in PBS using 100 µL/well, and incubated overnight at 4°C. Each well was then washed three times with 250 µL 0.1% Tween-20 in PBS to remove unbound rhGAA. Plates were blocked for 1 hour at room temperature using 150 µL/well 5% non-fat milk, 0.1% Tween-20 in PBS. Plates were then washed three times as described above, followed by the addition of 100 µL/well of serially diluted plasma samples (range 1∶100 to 1∶51000). Plates were incubated at 37°C for 1 hour, then washed as described above, followed by 1-hour incubation at room temperature with 100 µL/well of 1∶5000 diluted horseradish peroxidase-conjugated donkey anti-mouse IgG (ThermoPierce, Jackson Immunosearch Labs, West Grove, PA). Unbound secondary antibody was washed away, and 100 µL/well Turbo TMB ELISA substrate (Thermo Fisher Scientific) was added and incubated at room temperature for 5 to 10 minutes for color development. The reaction was stopped by the addition of 50 µL/well 1 M H2SO4 and absorbance was read at 450 nm on a Victor3 plate reader (Perkin Elmer, Waltham, MA). To determine IgG titers, an arbitrary cutoff value was set as 2-times the Abs450 nm level measured for blanks (defined as the value produced with Lysis Buffer only). The titer of each plasma sample was recorded as the last dilution factor with an Abs450 nm value that was greater than the cutoff value. Values represent the mean of 4 to 7 mice/group. T [file pone.0102092.s004.tif]
